# Predictors of adverse gambling behaviours amongst elite athletes

**DOI:** 10.1038/s41598-023-27469-8

**Published:** 2023-01-16

**Authors:** Matthew Adam Turk, Colm Murphy, Jack McCaffrey, Kieran Murray

**Affiliations:** 1 University College Dublin, Dublin, Ireland; 2 Washington Street Medical Centre, Cork, Ireland; 3 Children’s Health Ireland, Temple Street, Dublin, Ireland; 4 University Hospital Limerick, Dooradoyle, Limerick, Ireland

**Keywords:** Human behaviour, Risk factors

## Abstract

Problem gambling levels amongst elite sportspeople are above populational baseline. We assess gambling in an elite Irish sporting population. An anonymous web-based questionnaire including the validated Problem Gambling Severity Index was distributed. Univariate and multivariate analyses were performed to evaluate predictors of moderate/high risk gambling. 608 players (mean age 24) were included. Seventy nine percent of respondents were current gamblers and 6% problem gamblers. Amongst high-risk gamblers, significantly more were male (100% vs 76%, p = 0.003), fewer completed university (52% vs 69%, p = 0.024), and more were smokers (48% vs 24%, p = 0.002). They were also more likely to avail of free online gambling offers (90% vs 44%, p < 0.001), gamble with teammates (52% vs 21%, p < 0.001) and have placed their first bet before age 16 (41% vs 19%, p = 0.003). In multivariate analysis, moderate/high risk gambling was associated with: male gender (OR = 8.9 [1.1–69], p = 0.035), no 3rd level education (OR = 2.5 [1.4–5.0], p = 0.002), free online gambling use (OR = 4.3 [2.1–5.3], p < 0.001), gambling with teammates (OR = 3.0 [1.7–5.3], p < 0.001), and being under 18 at first bet (OR = 2.0 [1.1–3.3], p = 0.013). This study shows a harmful gambling culture amongst elite Irish athletes. Male gender, lower educational status, free online gambling use, gambling with teammates and first bet at less than age 18 were associated with moderate/high risk gambling. These groups may benefit from targeted interventions.

## Introduction

Gambling is a major public health concern. Problem gambling is gambling to a degree that compromises, disrupts or damages personal, family or recreational pursuits^1^. It is associated with mental health issues (anxiety and substance use problems) and psychosocial maladjustment (financial difficulties and social issues)^2,3^. One study of gambling treatment program attendees found 80% had suicidal ideations and 12% attempted suicide^4^. As such it presents a large societal burden.

Gambling includes a wide range of activities not limited to: casino games, slot machines, lotteries and online betting^5^. Prevalence of gambling problems varies across Europe, from 0.15% in Holland to 6.6% in Finland^6,7^. The prevalence of gambling problems is 0.8% in Ireland, but increases to 2.9% in males aged 25–34^8^. There remains no national treatment structure for problem gamblers^9^.

Fifty seven percent of British adults have gambled in the past year and 0.7% are problem gamblers^1,5^. Previous studies have shown a higher prevalence of problem gambling amongst athletes compared to the general population, with rates as high as 8.2%^10,11^. Online gambling is the industry’s largest growth area, with over 33 million active online gambling accounts in the UK^1^. 14% of UK 11–16 year olds have gambled in the past week^12^.

Gambling culture in sport has had problematic implications for athletes and society. It has been associated with altered results and match fixing, which compromises the integrity of sport^13^. There also exists a large discrepancy between the practicalities of corruption in sport due to gambling and players’ insight into that corruption^14^. Players have also been seen to bet on their own performance, which can be both detrimental to their athletic performance and mental health^15^. Gambling can also impact the players on a personal level; players have reported that gambling culture starts communally but evolves to happen in isolation and is emboldened by a culture that stigmatizes disclosure^16^. It can also have an exacerbating emotional impact on players, where players who are suffering from sport anxiety or depression are more likely to gamble than their colleagues^17^.

Founded in 1884, the Gaelic Athletic Association (GAA) oversees Gaelic football, hurling, handball and rounders^18^. The organisation aimed to make indigenous sports more accessible to the Irish population and there are now more than over 2200 clubs in all 32 counties of Ireland. With over 800,000 members, the GAA is Ireland’s major sporting organisation. Hurling and Gaelic football are high-intensity, multidirectional contact, amateur field sports^19^. With 1.5 million people attending the elite (intercounty) championships yearly prior to the Covid-19 pandemic, these are the best attended field sports in Ireland at elite level^20^. At 82,300, the assocation’s headquarters at Croke Park is amongst the largest stadia in Europe^18^.

The GAA’s Official Guide 2013 Rule 7.2(e) states match-fixing or improperly influencing the outcome of a game for your financial gain or another’s could fall under ‘Misconduct considered to have discredited the Association’^21^. Punishments range from an 8 week suspension to debarment and expulsion from the Association^21^. In 2018, the GAA prohibited a betting firm sponsoring any GAA competition, team, playing gear, or facility^22^. However, despite this legislation gambling remains an issue within the organisation with a number of high-profile elite Gaelic athletes having described their gambling addictions.

The GAA’s prohibition of gambling sponsorship is in sharp contrast to the “gamblification” of British sport. For the 2021–2022 season, 45% of English Premier League teams had a gambling company as their front-of-jersey sponsor^23^. The top tier of English rugby league is sponsored by the industry.

Much of Ireland’s legislation related to gambling was fragmented and outdated dating from the Totalisator Act (1929), the Betting Act (1931) and the Gaming and Lotteries Act (1956)^24^. However, in 2022 the transformative Gambling Regulation Bill repealed and replaced these and is expected to be enacted into law and operational by the end of 2023^25^. Amongst other restrictions, this Bill will ban gambling advertising between 5.30 a.m. and 9.00 p.m. on radio and television^25^.

The players’ representative bodies [Women’s Gaelic Players Association (WGPA) and Gaelic Players Association (GPA)] have a combined membership of around 3500^26^.

Sports sponsorship is a key facet of gambling marketing to connect gambling, sport and elite athletes. Image transfer may occur by linking gambling with sportspersons^27,28^. Advertising connecting gambling and sport may result in the ‘sanitation’ or “sportswashing” of gambling^29,30^. The symbolic linkage of sport and gambling can especially affect vulnerable groups including underage and problem gamblers^31^. Gambling may also lead to a misallocation of resources or an implicit sports tax on lottery products^32^.

This study explores gambling patterns in elite level Irish GAA athletes and investigates factors associated with higher risk gambling in this group.

## Methods

### Participants

Prior to distribution, the questionnaire was distributed to relevant stakeholders as a pilot (public health doctors, psychiatrists, a medical consultant, elite and non-elite GAA players) to ensure face validity, readability and that it was an appropriate length.

All current adult GPA and WGPA members were invited to complete the final survey. All current elite (senior intercounty) GAA players in Gaelic football/hurling/both who answered the question on gambling status were included for analysis. Exclusion criteria were not answering questions on playing or gambling status and being retired.

### Design

This point prevalence study assessed gambling behaviours and culture amongst elite GAA athletes. The questionnaire formed part of the survey entitled *Alcohol and Gambling in Intercounty GAA Players* as described previously^33^. The incentivized anonymous self-administered electronic SurveyMonkey™ questionnaire was distributed electronically to all adult GPA and WGPA members on 8th April 2019. In the event of non-response, the survey was resent once. Informed consent was obtained and all research carried out was in compliance with the declaration of Helsinki. Ethical approval for the study was approved by the clinical research ethics committee of the Cork Teaching Hospitals (reference number ECM 4 (gg) 04/12/18).

Demographic questions assessed age, gender, education, smoking status, marital status, employment, living arrangements, playing status, sport and playing level.

Current gambling was defined as having placed a bet within the last year. Data collected included frequency of each gambling activity, preferred method of gambling online (if applicable), age of first gambling experience, following a gambling company online. Frequency of different gambling activities was adopted from a previous survey with four response options: ‘never’, ‘less than once a month’, ‘1 to 4 times a month’ or ‘most days’^8^. These response options have been previously used in research related to online gambling^34^. In this current study, those who participated in an activity ‘most days’ or ‘1 to 4 times a month’, were considered regular gamblers with those answering ‘less than once a month’, or ‘never’ were considered non-regular gamblers for that activity.

Gambling severity was assessed with the nine item Problem Gambling Severity Index (PGSI) (questions 44–52). Published in 2011, the PGSI is a validated sub-set of the 31-item Canadian Problem Gambling Index^35^, which has been used in numerous national prevalence studies^36–38^. The PGSI divides gamblers into four risk categories: non- problem gambler (score = 0), low-risk gambler (1–2), moderate-risk gambler (3–7), problem gambler (8 or more). In our study, we classified problem gambling as those with moderate risk and above as the PGSI is best validated to identify problematic gambling behaviours in those with these scores. These scores reflect patients who spend more money than they can afford, feel guilty about their gambling, lose track of time gambling, and gamble to win back money that they have lost^39^. Question 58 was adapted from the South Oaks Gambling Screen to quantify the largest sum gambled on any 1 day (ranging from ≤ $1.00 to > $10,000)^40^. Given our study population, currency was changed from dollars to euro. Participants who had not gambled in the past 12 months were classified in the ‘no problem gambling’ category.

Gambling culture was assessed (age at first bet, following gambling companies online, gambling with teammates, knowledge re GAA gambling regulations and attitude to sponsorship). Respondents were encouraged to comment *on gambling in the GAA* and asked to whom they would turn for assistance gambling issues.

On finishing the survey, gambling support groups were listed. To increase response rate, respondents who finished the survey were given a code to possibly obtain a sports store or supermarket voucher.

### Statistical analysis

Categorical data are presented as the number (%). Normal data is presented as mean and standard deviation (SD). Non-normally distributed data are presented as median and interquartile range (IQR). Between group differences were analysed using unpaired 2-tailed t-tests, Mann–Whitney U test, chi-square or Fisher’s exact tests as appropriate.

Univariate analyses assesed for predictors moderate/high risk gambling (PGSI ≥ 3). Variables with p values < 0.1 in the univariate were entered into a multivariate logistic regression analysis model. Age was retained in the multivariate model despite not meeting statistical significance in the univariate model as is standard practice in epidemiological analyses given its importance as a confounder. Data analyses were performed using SPSS 27 IBM^®^.

### Ethical approval

Ethical approval was provided by the Clinical Research Ethics Committee of the Cork Teaching Hospitals.

### Patient and public involvement

This study was designed in consultation with the Gaelic Players Association (GPA) and Women’s Gaelic Players Association (WGPA) representative bodies. Prior to distribution, the questionnaire was distributed to elite and non-elite GAA players. Relevant gambling support groups were provided at the conclusion of the questionnaire. JM was an elite GAA footballer. Findings have been presented to the players representative bodies to help inform player supports. CM is team doctor to an elite hurling team.

## Results

### Respondent characteristics

773 players responded with a mean completion time of 9 min. Retired players (n = 54), those who did not clarify playing status (n = 2) or gambling status (n = 109) were excluded. Thus, 608 players were included in the study. Participant characteristics are shown in Table 1. Mean (SD) age was 24 (4) years. Most respondents were male, unmarried, non-smokers, in full-time employment, had completed university and were living with their parents. Roughly a third of players were in each of the top divisions and a third split between divisions 3 and 4. Seventy-nine percent (479/608) of respondents were current gamblers. A comparison of problem versus non-problem gamblers identified significant differences between the two groups (Table 1). Among high-risk gamblers, significantly more were male (100% vs 79%, p = 0.003), fewer completed university (52% vs 69%, p = 0.024), and more were smokers (48% vs 24%, p = 0.002). All 29 problem gamblers identified were male. The mean (SD) age of problem gamblers was 25 (4) years.

**Table 1:** Characteristics of study participants.

	Respondents	High risk gamblers	Not high risk gamblers	p value*
Male	460 (76%)	29 (100%)	344 (79%)	**0.003**
Mean (SD) age	24 (4)	25 (4)	24 (4)	0.296
Unmarried	559 (92%)	26 (90%)	317 (92%)	0.319
**Employment status**				
Full time employment	340 (56%)	18 (62%)	254 (58%)	0.348
Student	421 (40%)	9 (31%)	163 (37%)	0.244
Part time employed	22 (4%)	2 (7%)	14 (3%)	
Unemployed	5 (1%)	0 (0%)	3 (1%)	
**Educational status**				
Completed university	403 (66%)	15 (52%)	302 (69%)	**0.024**
Completed secondary	202 (33%)	14 (48%)	131 (30%)	
Completed primary only	4 (1%)	0 (0%)	2 (< 1%)	
**Smoking status**				
Non-smoker (past year)	476 (78%)	15 (52%)	331 (76%)	**0.002**
Less than daily	124 (20%)	13 (45%)	97 (22%)	
Daily	9 (2%)	1 (3%)	7 (2%)	
**Living arrangement**				
Parents	328 (54%)	13 (45%)	225 (52%)	0.236
Partner	121 (20%)	7 (24%)	90 (21%)	0.329
Inter-county teammates	38 (6%)	2 (7%)	31 (7%)	0.999
Other friends	101 (17%)	5 (17%)	75 (17%)	0.999
Other	21 (3%)	2 (7%)	14 (3%)	
**Playing status**				
Playing	546 (90%)	24 (83%)	389 (89%)	0.133
Injured	63 (10%)	5 (17%)	46 (11%)	
**Sports played at elite level**				
Gaelic football	322 (53%)	17 (59%)	225 (52%)	0.240
Hurling	273 (45%)	11 (38%)	201 (46%)	0.190
Both	13 (2%)	1 (3%)	8 (2%)	0.272
**Playing division**				
1	207 (34%)	9 (31%)	143 (33%)	0.419
2	192 (32%)	8 (28%)	139 (32%)	
3	117 (19%)	6 (21%)	89 (21%)	
4	93 (15%)	6 (21%)	64 (15%)	

*High risk gamblers versus not. Significant values are in bold.

### Problem Gambling Severity Index score

Median (IQR) PGSI score was 0 (IQR 0–1) amongst current (past year) gamblers. Of the current gamblers, 294 (63%) were non-problem gamblers (PGSI = 0), 86 (19%) were low risk gamblers (PGSI = 1–2), 55 (11.9%) were moderate risk gamblers (PGSI = 3–7) and 29 (6%) were problem gamblers (PGSI ≥ 8) (Table 2).

**Table 2:** Problem Gambling Severity Index amongst current gamblers.

PGSI category	Total	Male	Female	p (male to female)*
Non-problem gambler (PGSI = 0)	294 (63%)	216 (58%)	78 (86%)	**p < 0.001**
Low risk gambler (PGSI = 1–2)	86 (18%)	74 (20%)	12 (13%)	p = 0.072
Moderate risk gambler (PGSI = 3–7)	55 (12%)	54 (14%)	1 (1%)	**p < 0.001**
Problem gambler (PGSI ≥ 8)	29 (6%)	29 (8%)	0 (0%)	**p = 0.003**

*Values compared using a chi squared test. Significant values are in bold.

### Gambling behaviours

The most common method of gambling was on horse/dog racing. The relative frequency of different methods of gambling is shown in Table 3. 47% have availed of free online gambling offers in the past. Placing a bet at a horse/dog race was the most frequent mode of gambling. Analysis of males in this category shows 17% never bet this way, 58% less than once per month, 17.5% one to four times per month and 7% most days. Analysis of females in this category shows 17% never bet this way, 81% less than once per month, 2% one to four times per month and 0% most days, showing a decreased frequency of betting in the most common media in women.

**Table 3:** Forms of gambling within the past year amongst current gamblers.

Form of gambling	Total
At a horse/dog race meeting	383 (83%)
In a bookmakers	355 (77%)
Lottery ticket/scratch card	352 (76%)
Online/telephone	273 (59%)
Card games for money with family/friends	256 (55%)
At a casino	204 (44%)
Gaming/slot machines	146 (31%)
Bingo	118 (25%)
Lottery game online	82 (17%)

Compared to lower risk gamblers, Problem gamblers were more likely to avail of free online gambling offers (90% vs 44%, p < 0.001) and gamble with intercounty (elite) teammates (52% vs 21%, p < 0.001 and to have placed their first bet before 16 years of age (41% vs 19%, p = 0.003).

Fourteen participants (2%) had gambled over €1000 in 1 day and 2 (< 1%) more than €10,000 in any 1 day. Ten percent of high risk gamblers placed €1,000 or greater bets in 1 day compared to 1% of lower risk gamblers (p < 0.001). It is worth noting that these players are amateur athletes. Non-problem gamblers are more likely than problem gamblers to have a maximum daily bet of less than €100 (48% vs 7%, P < 0.001).

Mobile ‘app’ on a phone (290/463 participants—63%) was the most common device used to gamble online, followed by laptop (32 participants—7%), tablet (15 participants—3%) and desktop (13 participants—3%) (Table 4). Twitter was the most common social media platform to follow a gambling company online (n = 189, 41%). This was followed by Facebook (n = 125, 27%), followed by Instagram (n = 96, 21%), Snapchat (n = 75, 16%), and youtube (n = 8, 2%).

**Table 4:** Online gambling habits amongst current gamblers.

**Devices used**	
Mobile application on phone	290 (63%)
Laptop	32 (7%)
Tablet	15 (3%)
Desktop	13 (2%)
None	170 (37%)
Types of device, median (IQR)	1.0 (0.0–1.0)
**Follow a gambling company on the following online platforms**	
Twitter	189 (41%)
Facebook	125 (27%)
Instagram	96 (21%)
Snapchat	75 (16%)
Other (Youtube/Google + etc.)	15 (3%)
None	197 (43%)
Types of platforms, median (IQR)	1.0 (0.0–2.0)

### Gambling culture

Of the participants, 107 (23%) of the total sample gamble with intercounty (elite) team-mates. Twenty eight percent felt it was appropriate for the gambling industry to sponsor GAA competitions. Worryingly, only 487 (81%) participants were aware it is against the GAA rules to bet on a game in which you are involved. Just over half of participants placed their first bet before aged 18 (Table 5).

**Table 5:** Gambling behaviours.

	Respondents	Problem gamblers	Not problem gamblers	p value*
Availed of free online gambling offers	217 (47%*)	29 (90%)	191 (44%)	** < 0.001**
Gambled with intercounty (elite) teammates	107 (23%*)	15 (52%)	92 (21%)	** < 0.001**
Mean (SD) age at first bet	17.0 (2.7)			
**Age at first bet**				
< 16	104 (21%)	12 (41%)	82 (19%)	**0.003**
16–17	152 (30%)	9 (31%)	127 (30%)	
> 17	251 (49%)	8 (28%)	205 (47%)	
Aware illegal to bet on a game you are involved in	487 (81%)	23 (80%)	365 (84%)	0.259
Feel appropriate for gambling sponsorship of GAA competitions	166 (28%)	9 (31%)	136 (31%)	0.49
**Largest amount gambled in 1 day**				
Never gambled	82 (14%)	0	18 (4%)	0.207
< €1	13 (2%)	1 (3%)	7 (2%)	0.617
€1.01–€10.00	171 (29%)	3 (10%)	133 (31%)	**0.002**
€10.01–€100.00	224 (38%)	2 (7%)	207 (48%)	** < 0.001**
€100.01–€1000.00	84 (14%)	13 (44%)	66 (15%)	**0.001**
€1000.01–€10,000.00	14 (2%)	10 (35%)	3 (1%)	** < 0.001**
> €10,000.00	2 (< 1%)	0 (0%)	0 (0%)	

*Of current (past year) gamblers. Significant values are in bold.

If they had issues with alcohol or gambling, the most common source of support chosen was family (35%), followed by friends other than intercounty (elite) team-mates (20%), partner (16%) and players representative bodies (16%). Only 6% would turn to doctor or counsellor and 4% to an intercounty (elite) teammate.

### Associations with moderate/problem gambler (PGSI ≥ 3)

In multivariate binary logistic regression analysis, moderate/high risk gambling was associated with: male gender (OR = 8.9 [1.1–69], p = 0.035), no 3rd level education (OR = 2.5 [1.4–5.0], p = 0.002), free online gambling use (OR = 4.3 [2.1–5.3], p < 0.001), gambling with teammates (OR = 3.0 [1.7–5.3], p < 0.001), and being under 18 at their first bet (OR = 2.0 [1.1–3.3], p = 0.013) (Tables 6 and 7).

**Table 6:** Univariate analysis for moderate/high risk gambling (PGSI ≥ 3).

Variable	OR (95%CI)	p
Male	25.7 (3.5–187.6)	**0.001**
Age < 25 years old	1.2 (0.8–1.9)	0.429
Single	1.2 (0.5–3.1)	0.646
Living with partner	0.7 (0.4–1.3)	0.293
Full time employed	0.7 (0.5–1.2)	0.200
Student	1.1 (0.7–1.9)	0.475
Not completed university	2.0 (1.4–3.3)	**0.002**
Smoker	2.1 (1.2–3.5)	**0.004**
Top 2 divisions	0.9 (0.6–1.5)	0.931
Football only	1.1 (0.7–1.8)	0.613
Injured (versus playing)	1.6 (0.8–3.2)	0.150
Availed of free online gambling offers	6.6 (3.7–11.7)	** < 0.001**
Gamble with inter-county teammates	5.2 (3.1–8.6)	** < 0.001**
Approve of gambling sponsorship of GAA competitions	1.5 (0.9–2.4)	0.136
Adverse alcohol use	3.5 (1.5–8.4)	**0.005**
Follow a gambling company online	2.6 (1.5–4.4)	** < 0.001**
Under 18 at first bet	2.5 (1.7–5.0)	** < 0.001**
Alcohol in past year	3.9 (0.5–29.6)	0.190
Monthly or more frequent binge drinker	1.3 (0.8–2.2)	0.218

Significant values are in bold.

**Table 7:** Multivariate analysis for moderate/high risk gambling (PGSI ≥ 3).

Variable	OR (95%CI)	p
Male	8.9 (1.1–69.0)	**0.035**
Availed of free online gambling offers	4.3 (2.1–8.6)	** < 0.001**
Gamble with inter-county teammates	3.0 (1.7–5.3)	** < 0.001**
Not completed university	2.5 (1.4–5.0)	**0.002**
Under 18 at first bet	2.0 (1.1–3.3)	**0.013**
Smoker	1.4 (0.8–2.5)	0.309
Follow a gambling company online	1.0 (0.5–2.0)	0.908
Adverse alcohol use	0.9 (0.5–1.6)	0.655

Significant values are in bold.

## Discussion

### Main findings

Despite the ban on gambling sponsorship on any GAA activities, there is worrying gambling behaviours amongst elite Gaelic athletes. As in other elite sportspeople, levels of problem gambling amongst elite GAA players are above populational baseline^10,11^.

Seventy nine percent of players were current gamblers. This is higher than both the general Irish population (65%) and European professional athletes (57%)^8,10^. This study highlights a problem gambling rate of 4.8% in this population, compared to 0.8% for the general population of Ireland. In Ireland, the prevalence of problem gambling in males aged 25–34 is 2.9% and 1.9% in males aged 18–24^8^. The prevalence of current problem gambling in this study is less than that reported in professional European athletes (8.2%), current or past and elite Swedish athletes (7%)^10,11^.

In multivariate analysis, male gender, no 3rd level education, free online gambling use, gambling with teammates, and being under 18 at their first bet were associated with a player’s moderate to high risk gambling. With limited availability of support services, interventions targeting these groups may be an efficient approach. Some of these results are consistent with the established literature. In our univariate analysis, male gender had an odds ratio of 25.7 for being associated with problem gambling. This compares similarly to a Swedish study from 2018 that reported an odds ratio of 11.4, albeit in a cohort of far fewer patients^11^. It appears male gender is associated with problem gambling regardless of the sports club studied. An American study looking at student athletes found odds ratios of 3 for increased gambling frequency in male compared to female athletes^41^.

Among the total sample, 59% of participants had gambled on the internet or a mobile phone in the past 12 months. It has been shown in the past that internet gamblers are three to four times more likely to develop a gambling problem^42^.

Most players would turn to family (35%) or friends (20%) for help with addiction issues. Given this finding, it is important these groups are engaged.

Many players recognise the harmful gambling culture within elite GAA. For example, “Biggest problem in the GAA. I have had problems in the past which required me to seek help for gambling addiction.” and “Gambling is a huge problem within GAA teams. I am a compulsive gambler myself…in training dressing rooms or on buses to matches it is the core of the conversation for a high percentage of players. I feel it needs to be addressed further and should be seen in the public eye as a serious issue.” In a 2017 GPA survey, 23% of elite male GAA players felt there is a gambling problem within the GAA^43^.

Interestingly, some players believe that elite athletes personality type lends itself to gambling issues:

“The addictive/obsessive nature of most inter county player's personalities leave them very susceptible to gambling problems. With a feeling of no outlet, online gambling can be the 'buzz' that this type of person seeks.” and “I've seen the impact on a daily basis gambling can have on individuals and with inter county players being so driven I can only imagine the impact gambling will have on their probably somewhat addictive personality”.

Worryingly, players also appear to be gambling on matches in their sport: “Most players bet on G.A.A due the knowledge they have on it.” and “Betting on club matches players seem to think they have better knowledge than the bookie especially in club championships and tournaments with little exposure”.

### Practical implications

Gambling has a significant financial and social impact on our society. The social cost of gambling in the UK ranges between £200 million and £1.2 billion^44^. Sociological harms to society caused by gambling may be as significant as those caused by alcohol abuse and major depressive disorders^45^. Recent years have seen strategies to curb problem gambling in Ireland. In 2018, the GAA prohibited betting firms sponsoring any competition, team, playing gear, or facility^22^. This action contrasts starkly with other sports such as English football. The GPA launched a phone line counselling service to deal with issues such as alcohol and gambling and released *Gambling a guide for county players & our games*^43^.

Improving health behaviours in athletes can be influential in wider society, as sportspeople can function as role models, especially to young people^46–48^. Premiership footballer Marcus Rashford’s successful movement to provide free school meals for socioeconomically deprived children in the UK showed how athletes can have a positive impact^49^. With over 14 million followers on Instagram and Twitter, Rashford has a wide following. Influencing elite athletes who in turn influence others could be a cost-effective and worthwhile approach to promote positive health outcomes.

Changing the harmful gambling culture associated with sports likely requires a multifaceted approach involving the athletes themselves, friends and family, players, representative bodies, backroom staff, government and sporting organisations and wider society. Thankfully, there are numerous evidence based solutions to address this. These include banning sports sponsorship by the gambling industry, limiting access to gambling for minors, increasing taxation on the gambling industry, screening and brief interventions and inpatient treatment facilities for individuals with addiction issues^50,51^.

Like with other industries such as alcohol and ultra-processed foods, powerful lobbying interests support marketing that encourages harmful and addictive behaviour. Evidence shows that these industries are ineffective at regulating themselves^52–54^ and healthcare inputs are required to ensure their effective regulation and to prevent significant societal harm.

Advertising plays a role in the development of concerning gambling behaviour through a variety of mechanisms. It can encourage a non-problem gambler to gamble to an extent that it becomes problematic. It can furthermore encourage people to engage with different forms of gambling. Studies have shown that an increased number of gambling platforms used is linked to decreased inhibition when gambling, which can contribute to pre-existing gambling problems^55^. Advertising can also make gambling seem more socially acceptable and encourage a broader audience to participate^56^.

This theory holds especially true for people in vulnerable populations, including young people. This study suggests a relationship between gambling as a minor and developing a gambling problem later in life. This finding is consistent with previous work that showed problem-gamblers having started gambling 8 years younger to their control counterparts^57^. Controlling gambling access to minors may be prevent a gambling addiction later in life. There were also associations between a lower level of educational achievement and higher-risk gambling. Completion of tertiary education has been commonly used as a surrogate marker for socioeconomic status, which points to the need to target these groups for gambling-related interventions^58^.

Access to interventions for those who need help may help alleviate the burden of problem gambling. Short cognitive behavioural therapy sessions may be beneficial^50^. Less intensive interventions have also shown to be beneficial. These include motivational phone calls, and professionally-directed journals or self-help books^51^.

The medical community is increasingly recognizing certain behavioural addictions to be similarly harmful to substance-abuse addiction. For example, gambling addiction was included in the Diagnostic and Statistical Manual of Mental Disorders (DSM–5)^59^. Dopamine, impulse control, and other neurochemical differences have been noted in patients suffering with gambling addiction^60^. As such, there is a need for psychiatric support for these patients.

### Methodological considerations and limitations

This study gives an in-depth analysis of gambling culture in elite Irish athletes. The survey was sent to every registered adult player preventing bias. The extensive cohort (n = 773) includes females, who have been under-represented in prior studies on this topic. Validated tools enable comparison with existing data. In addition to this quantitive data we also present qualitive findings concerning gambling culture and sources of support. This information is crucial to provide effective interventions. It would also be interesting in future work to analyse the types of sports athletes were gambling on, and if their gambling behaviours were limited to sport alone or included other means of gambling such as casino games.

Limitations of self-reported outcomes include the possibility of denial and repression^61^. Yet, self-report surveys have been validated and are regularly utilised to examine alcohol and gambling behaviours^37,38^.

## Conclusion

This study shows a harmful gambling culture amongst elite Irish athletes. All problem gamblers were male. Male gender, lower educational status, free online gambling use, gambling with teammates, and being aged 18 or less when placing your first bet were associated with moderate/high risk gambling. Targeting individuals with these characteristics may be a cost-effective approach for gambling interventions. Educating partners and family members about addiction issues is important, as currently this is who the players are most likely to ask for help.

## Data Availability

The dataset used and analysed during the current study is available from the corresponding author on reasonable request.

## References

[1] Conolly, A., Fuller, E., Jones, H., Maplethorpe, N., Sondaal, A. & Wardle, H. Gambling behaviour in Great Britain in 2015. Evidence from England, Scotland and Wales London: National Centre for Social Research (2017).

[2] Cowlishaw S, Kessler D (2016). Problem gambling in the UK: Implications for health, psychosocial adjustment and health care utilization. Eur. Addict. Res..

[3] Black DW, McCormick B, Losch ME, Shaw M, Lutz G, Allen J (2012). Prevalence of problem gambling in Iowa: Revisiting Shaffer's adaptation hypothesis. Ann. Clin. Psychiatry.

[4] McCormick RA, Russo AM, Ramirez LF, Taber JI (1984). Affective disorders among pathological gamblers seeking treatment. Am. J. Psychiatry.

[5] Wardle H, Reith G, Langham E, Rogers RD (2019). Gambling and public health: We need policy action to prevent harm. BMJ.

[6] Goudriaan AE (2014). Gambling and problem gambling in the N etherlands. Addiction.

[7] Castrén S, Basnet S, Pankakoski M, Ronkainen J-E, Helakorpi S, Uutela A (2013). An analysis of problem gambling among the Finnish working-age population: A population survey. BMC Public Health.

[8] Alcohol NACoDa. Prevalence of Drug Use and Gambling in Ireland and Drug Use in Northern Ireland: National Advisory Committee on Drugs and Alcohol (NACDA) (2016) https://health.gov.ie/wp-content/uploads/2016/11/Bulletin-1.pdf (Accessed 27 July 2019).

[9] O’Gara C (2018). The gambling control bill: Time for action. Irish J. Psychol. Med..

[10] Grall-Bronnec M, Caillon J, Humeau E, Perrot B, Remaud M, Guilleux A (2016). Gambling among European professional athletes. Prevalence and associated factors. J. Addict. Dis..

[11] Håkansson A, Kenttä G, Åkesdotter C (2018). Problem gambling and gaming in elite athletes. Addict. Behav. Rep..

[12] Young People & Gambling 2018. Gambling Commission Contract No.: 28 July (2018).

[13] Şeker FS, Şahin M (2018). The communal effects of bet match fixing in football. Eur. J. Educ. Stud..

[14] Lastra R, Bell P, Bond C (2018). Sports betting and the integrity of Australian sport: Athletes' and non-athletes’ perceptions of betting-motivated corruption in sport. Int. J. Law Crime Justice..

[15] Moriconi M, de Cima C (2020). Betting practices among players in Portuguese championships: From cultural to illegal behaviours. J. Gambl. Stud..

[16] Lim MSM, Bowden-Jones H, Salinas M, Price J, Goodwin GM, Geddes J (2017). The experience of gambling problems in British professional footballers: A preliminary qualitative study. Addict. Res. Theory..

[17] Jensen SN, Ivarsson A, Fallby J, Elbe A-M (2019). Gambling behaviors among Danish and Swedish elite football players. J. Clin. Sport Psychol..

[18] Association GA. About the GAA. https://www.gaa.ie/the-gaa/about-the-gaa/ (Accessed 27 Feb 2019).

[19] McIntyre M (2005). A comparison of the physiological profiles of elite Gaelic footballers, hurlers, and soccer players. Br. J. Sports Med..

[20] Gouttebarge V, Tol JL, Kerkhoffs GM (2016). Epidemiology of symptoms of common mental disorders among elite Gaelic athletes: A prospective cohort study. Phys. Sportsmed..

[21] Association GA. *Problem Gambling—A Growing Concern in Modern Ireland*.

[22] Association GA. GAA Launch Gambling Awareness Campaign ‘Reduce the Odds’ 2018 (updated 9 August 2018) https://www.gaa.ie/news/gaa-launch-gambling-awareness-campaign-reduce-the-odds/ (Accessed 5 Mar 2019).

[23] Koeshartanto, M. 2021–2022 Premier League Jersey Sponsors: Gilted Edge Soccer Marketing (2021) https://www.giltedgesoccer.com/2021-22-premier-league-jersey-sponsors/ (Accessed 7 Dec 2021).

[24] Oireachtas Hot. Gambling Regulation Bill 2022 (2022) https://www.oireachtas.ie/en/bills/bill/2022/114/ (Accessed 9 Dec 2022).

[25] Oireachtas Hot. Gambling Regulation Bill 2022 (2022).

[26] Elish Kelly, J. B., McGuinness, S. & Watson, D. Playing Senior Inter-county Gaelic Games: Experiences, Realities and Consequences (2018). https://www.gaelicplayers.com/Portals/0/Publications/RS76.pdf (Accessed 1 Mar 2019).

[27] Chen C-Y, Lin Y-H, Claussen CL (2012). Celebrity endorsement for sporting events using classical conditioning. Int. J. Sports Mark. Spons..

[28] Keller KL (1993). Conceptualizing, measuring, and managing customer-based brand equity. J. Mark..

[29] McMullan JL, Miller D (2008). All in! The commercial advertising of offshore gambling on television. J. Gambl. Issues..

[30] Milner L, Hing N, Vitartas P, Lamont M (2013). Embedded gambling promotion in Australian football broadcasts: An exploratory study. Commun. Polit. Cult..

[31] Lopez-Gonzalez H, Griffiths MD (2017). Betting, forex trading, and fantasy gaming sponsorships—a responsible marketing inquiry into the ‘Gamblification’ of english football. Int. J. Ment. Health Addict..

[32] Forrest D, Simmons R (2003). Sport and gambling. Oxf. Rev. Econ. Policy.

[33] Murray K, Murphy C, Herlihy A, McCaffrey J, Codd M, Murray FE (2021). Harmful alcohol consumption in elite sports players in Ireland. Irish J. Med. Sci. (1971).

[34] McCormack A, Shorter GW, Griffiths MD (2013). An examination of participation in online gambling activities and the relationship with problem gambling. J. Behav. Addict..

[35] Ferris JA, Wynne HJ (2001). The Canadian Problem Gambling Index.

[36] Wardle H, Moody A, Griffiths M, Orford J, Volberg R (2011). Defining the online gambler and patterns of behaviour integration: Evidence from the British Gambling Prevalence Survey 2010. Int. Gambl. Stud..

[37] Devlin ME, Walton D (2012). The prevalence of problem gambling in New Zealand as measured by the PGSI: Adjusting prevalence estimates using meta-analysis. Int. Gambl. Stud..

[38] Barbaranelli C, Vecchione M, Fida R, Podio-Guidugli S (2013). Estimating the prevalence of adult problem gambling in Italy with SOGS and PGSI. J. Gambl. Issues..

[39] Miller NV, Currie SR, Hodgins DC, Casey D (2013). Validation of the problem gambling severity index using confirmatory factor analysis and rasch modelling. Int. J. Methods Psychiatr. Res..

[40] Lesieur HR, Blume SB (1987). The South Oaks Gambling Screen (SOGS): A new instrument for the identification of pathological gamblers. Am. J. Psychiatry.

[41] Weinstock J, Whelan JP, Meyers AW, Watson JM (2007). Gambling behavior of student-athletes and a student cohort: What are the odds?. J. Gambl. Stud..

[42] Wood RT, Williams RJ (2011). A comparative profile of the Internet gambler: Demographic characteristics, game-play patterns, and problem gambling status. New Media Soc..

[43] Association GP. Gambling a guide for county players & our games (Gaelic Players Associtation, 2017) https://www.gaelicplayers.com/Portals/0/SocialResponsibility/0270_gambling_document_online.pdf.

[44] Research IfPP. Cards on the Table: the Cost to Government associated with people who are problem gamblers in Briatin (2016). https://www.ippr.org/files/publications/pdf/Cards-on-the-table_Dec16.pdf (Accessed 19 Jan 2022).

[45] Foundation VRG. Assessing Gambling-related Harm in Victoria: A Public Health Perspective. https://responsiblegambling.vic.gov.au/resources/publications/assessing-gambling-related-harm-in-victoria-a-public-health-perspective-69/ (Accessed 19 Jan 2022).

[46] Bandura A, Walters RH (1977). Social Learning Theory.

[47] Armour K, Duncombe R (2012). Changing lives? Critical evaluation of a school-based athlete role model intervention. Sport Educ. Soc..

[48] Bush AJ, Martin CA, Bush VD (2004). Sports celebrity influence on the behavioral intentions of generation Y. J. Advert. Res..

[49] Sinha IP, Lee AR, Bennett D, McGeehan L, Abrams EM, Mayell SJ (2020). Child poverty, food insecurity, and respiratory health during the COVID-19 pandemic. Lancet Respir. Med..

[50] Petry NM, Weinstock J, Ledgerwood DM, Morasco B (2008). A randomized trial of brief interventions for problem and pathological gamblers. J. Consult. Clin. Psychol..

[51] Hodgins DC, Currie S, El-Guebaly N, Peden N (2004). Brief motivational treatment for problem gambling: A 24-month follow-up. Psychol. Addict. Behav..

[52] Noel JK, Babor TF (2017). Does industry self-regulation protect young people from exposure to alcohol marketing? A review of compliance and complaint studies. Addiction.

[53] Noel J, Lazzarini Z, Robaina K, Vendrame A (2017). Alcohol industry self-regulation: Who is it really protecting?. Addiction.

[54] O'Brien P, Stockwell T, Vallance K, Room R (2021). WHO should not support alcohol industry co-regulation of public health labelling. Addiction.

[55] Phillips JG, Ogeil R, Chow YW, Blaszczynski A (2013). Gambling involvement and increased risk of gambling problems. J. Gambl. Stud..

[56] Binde, P. Gambling advertising: A critical research review. 10.11575/PRISM/9519 (2014).

[57] Kessler RC, Hwang I, LaBrie R, Petukhova M, Sampson NA, Winters KC (2008). DSM-IV pathological gambling in the National Comorbidity Survey Replication. Psychol. Med..

[58] Herndon JE, Kornblith AB, Holland JC, Paskett ED (2013). Effect of socioeconomic status as measured by education level on survival in breast cancer clinical trials. Psychooncology..

[59] Regier DA, Kuhl EA, Kupfer DJ (2013). The DSM-5: Classification and criteria changes. World Psychiatry.

[60] Yau YH, Potenza MN (2015). Gambling disorder and other behavioral addictions: Recognition and treatment. Harv. Rev. Psychiatry..

[61] Jacobson E (1957). Denial and repression. J. Am. Psychoanal. Assoc..

